# Donor-Derived Cell-Free DNA for the Detection of Heart Allograft Injury: The Impact of the Timing of the Liquid Biopsy

**DOI:** 10.3389/ti.2022.10122

**Published:** 2022-03-21

**Authors:** Jeroen G. H. P. Verhoeven, Dennis A. Hesselink, Annemiek M. A. Peeters, Evert de Jonge, Jan H. von der Thüsen, Ron H. N. van Schaik, Maja Matic, Carla C. Baan, O. C. Manintveld, Karin Boer

**Affiliations:** ^1^ Division of Nephrology and Transplantation, Department of Internal Medicine, University Medical Center Rotterdam, Rotterdam, Netherlands; ^2^ Erasmus MC Transplant Institute, University Medical Center Rotterdam, Rotterdam, Netherlands; ^3^ Department of Clinical Chemistry, University Medical Center Rotterdam, Rotterdam, Netherlands; ^4^ Department of Pathology, University Medical Center Rotterdam, Rotterdam, Netherlands; ^5^ Department of Cardiology, Erasmus MC, University Medical Center Rotterdam, Rotterdam, Netherlands

**Keywords:** liquid biopsy, ddcfDNA, cfDNA, heart transplantation, endomyocardial biopsy

## Abstract

**Background:** In heart transplant recipients, donor-derived cell-free DNA (ddcfDNA) is a potential biomarker for acute rejection (AR), in that increased values may indicate rejection. For the assessment of ddcfDNA as new biomarker for rejection, blood plasma sampling around the endomyocardial biopsy (EMB) seems a practical approach. To evaluate the effect of the EMB procedure on ddcfDNA values, ddcfDNA values before the EMB were pairwise compared to ddcfDNA values after the EMB. We aimed at evaluating whether it matters whether the ddcfDNA sampling is done before or after the EMB-procedure.

**Methods:** Plasma samples from heart transplant recipients were obtained pre-EMB and post-EMB. A droplet digital PCR method was used for measuring ddcfDNA, making use of single-nucleotide polymorphisms that allowed both relative quantification, as well as absolute quantification of ddcfDNA.

**Results:** Pairwise comparison of ddcfDNA values pre-EMB with post-EMB samples (*n* = 113) showed significantly increased ddcfDNA concentrations and ddcfDNA% in post-EMB samples: an average 1.28-fold increase in ddcfDNA concentrations and a 1.31-fold increase in ddcfDNA% was observed (*p* = 0.007 and *p* = 0.03, respectively).

**Conclusion:** The EMB procedure causes iatrogenic injury to the allograft that results in an increase in ddcfDNA% and ddcfDNA concentrations. For the assessment of ddcfDNA as marker for AR, collection of plasma samples before the EMB procedure is therefore essential.

## Graphical Abstract

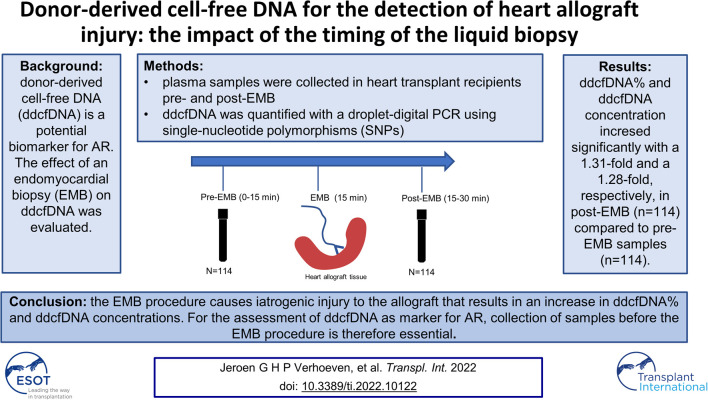

## Introduction

Heart transplant recipients are monitored for acute rejection (AR) by a strict endomyocardial biopsy (EMB) surveillance scheme. Histopathological examination of an EMB is currently the gold standard for diagnosing AR. However, this procedure is invasive, costly and can result in several complications, including coronary artery fistula formation and tricuspid regurgitation (1). Moreover, the diagnosis of AR may be missed as a result of sampling error. Finally, considerable variability exists in the interpretation of an EMB between pathologists (2). There is thus an unmet need for minimally-invasive biomarkers to timely diagnose heart transplant rejection.

Donor-derived cell-free DNA (ddcfDNA) is a promising biomarker that could improve AR monitoring in heart transplant recipients (3–7). ddcfDNA is highly fragmented DNA derived from apoptotic and necrotic cells (8). Based on genetic differences between the donor and recipient, such as single nucleotide polymorphisms (SNPs) or insertion and deletion variations of DNA sequences, it is possible to specifically detect donor cfDNA in blood plasma in a background of recipient cfDNA. The release of ddcfDNA especially occurs at times of allograft injury, including AR. Increased values of ddcfDNA were observed during high-grade heart transplant rejection (3, 4, 6, 7).

An EMB procedure itself also causes allograft injury that may result in an increase in ddcfDNA. Therefore, it is important to establish whether the timing of sampling is important for the interpretation of the ddcfDNA values.

DdcfDNA can be quantified as fraction (% ddcfDNA of total cfDNA) or as absolute concentration (copies/ml plasma). So far, in heart transplant recipients, studies mainly focussed on ddcfDNA% and not on concentration. An important limitation of ddcfDNA% is that values may be affected by fluctuations in recipient cfDNA, the denominator in the calculation of ddcfDNA%. These fluctuations in recipient cfDNA occur both during physiological conditions (9, 10), as well as pathological conditions, including infection and cancer (11, 12), that occur frequently in heart transplant recipients (13). For this reason, using ddcfDNA concentration might be more accurate to avoid the variability of ddcfDNA% (14). Additionally, the EMB procedure might not only affect the level of donor cfDNA but also of the recipient cfDNA. This implies that a potential effect of an EMB procedure on ddcfDNA% might be different in magnitude than for ddcfDNA concentration. Therefore, it is important to assess both values.

This present study aims 1) to determine the effect of the EMB procedure on plasma ddcfDNA and; 2) to assess both ddcfDNA% and ddcfDNA concentration (not subject to fluctuations in recipient cfDNA).

## Materials and Methods

### Study Design

Adult heart transplant recipients who were scheduled for an EMB were eligible for participation in this clinical study that was performed at the Erasmus MC, University Medical Center, Rotterdam, the Netherlands. The study was approved by the institutional review board of the Erasmus MC (Medical MEC-Review Board number 2017-196) and recipients gave written informed consent prior to participation. The study was conducted in accordance with the principles of the Declaration of Helsinki, consistent with the Good Clinical Practice guidelines of the International Conference on Harmonization.

### Clinical Sample Collection and Processing

Blood samples were collected from heart transplant recipients who underwent routine surveillance EMB. Samples were collected immediately before (<15 min pre-biopsy) and immediately after the biopsy procedure (<15 min post-biopsy). The EMB was performed via the jugular vein with a bioptome size of 7 French. In the early post-transplant phase, routine EMB was performed weekly for the first 2 months, monthly for the next 4 months, and then every 3 months.

Ten milliliters of blood was collected in anti-coagulated CellSave blood collection tubes (Menarini, Florence, Italy). Samples were stored at 4°C within 3 h after collection. The plasma was separated by centrifugation at 1,600 × g for 20 min within 24 h after collection, and stored at −30°C.

### DNA Isolation and Single Nucleotide Polymorphism Genotyping

Genomic DNA from recipients was obtained from peripheral blood mononuclear cells, and DNA from their corresponding donor was obtained from either spleen cells or heart transplant tissue (collected with routine surveillance of transplant rejection from an EMB) by automated purification (Maxwell, Promega, Leiden, Netherlands). According to Dutch law, spleen cells are considered as left over material. Therefore, for the use of these spleen cells, no informed consent of donors was necessary. Recipients and donors were genotyped by using an in house designed panel of 10 preselected SNPs by a quantitative PCR (Applied Biosystems™ QuantStudio™, Foster City, CA, United States). Per patient, one to three discriminative SNPs were selected for ddcfDNA quantification.

### cfDNA Isolation and Donor-Derived Cell-Free DNA Measurement

cfDNA was isolated from 3 ml of anti-coagulated blood plasma by using the Circulating Nucleic Acid kit (Qiagen, Hilden, Germany) according to the manufacturer’s instructions. The QX100 droplet digital PCR (ddPCR) system (Bio-Rad Laboratories, CA, United States) was used for the quantification of (dd)cfDNA. Samples of 20 μl were prepared for PCR reactions by making a mixture containing purified cfDNA, water, a donor specific target assay (discriminative SNP) and ddPCR Supermix for Probes (Bio-Rad). Droplets were generated with a QX100 droplet generator (Bio-Rad) according to the manufacturer’s instructions. The ddPCR was performed using the T100^TM^ Thermal Cycler (Bio-Rad) with the following amplification protocol: 95°C for 10 min, 40× (94° for 30 s, 55° for 1 min), then 98°C for 10 min. The quantified droplets were analyzed through a QX100 droplet reader (Bio-Rad) using Quantasoft software version 1.0.596 (Bio-Rad). ddcfDNA values were quantified either as fraction (%) (donor-specific SNP signal/total SNP signal (donor-specific SNP signal + non-donor-specific SNP signal)) or as concentration (copies/ml plasma). In samples were ddcfDNA was quantified with two or three SNPs, the ddcfDNA values were averaged.

### Biopsy Examination

All biopsies were examined and scored according to the ISHLT grading system by an experienced transplant pathologist (JvT) (15, 16). Biopsies were classified as acute cellular rejection (ACR) grade 0R–2R and as antibody-mediated rejection (pAMR) grade 0–2.

### Statistical Analysis

The primary objective of this study was to assess the effect of the EMB procedure on ddcfDNA values. IBM SPSS version 25 (Armonk, NY, United States) was used for statistical analysis of the data and for making the figures. Continuous variables are presented as median with interquartile range (first and third, IQR) for non-normally distributed data. Nonparametric data of paired samples before and after biopsy were compared pairwise using the Wilcoxon matched-pairs signed rank test. Results were considered statistically significant for two sided *p*-values below 0.05.

## Results

### Patients and Samples

A total of 226 paired samples from 15 patients (aged 18–63 years) was collected both pre-EMB (*n* = 113) and post-EMB (*n* = 113) between November 2019 and August 2020. The paired samples was collected between day 7 and day 509 post-transplant. Patient characteristics and an overview of the biopsy results are depicted in Tables 1, 2. An overview of the timing of the EMB biopsies with the ddcfDNA values and biopsy results is presented in Supplementary Figures 1A,B.

**TABLE 1:** Baseline characteristics.

Baseline characteristics	Study population (*n* = 15)
Patients	15
Age (years)	49 (18–63)
Female/Male	6 (40.0%)/9 (60.0%)

Continues variables are described as mean (range). Categorical variables as number of cases (%).

**TABLE 2:** Biopsy results.

Biopsy result and classification	Biopsies (*n* = 113)
ACR 0, ACR 1, AMR 0	111
ACR 2	2
AMR 2	0

Abbreviations: ACR, acute cellular rejection; AMR, antibody-mediated rejection.

### Effect of Endomyocardial Biopsy Procedure on Donor-Derived Cell-Free DNA

**FIGURE 1:**
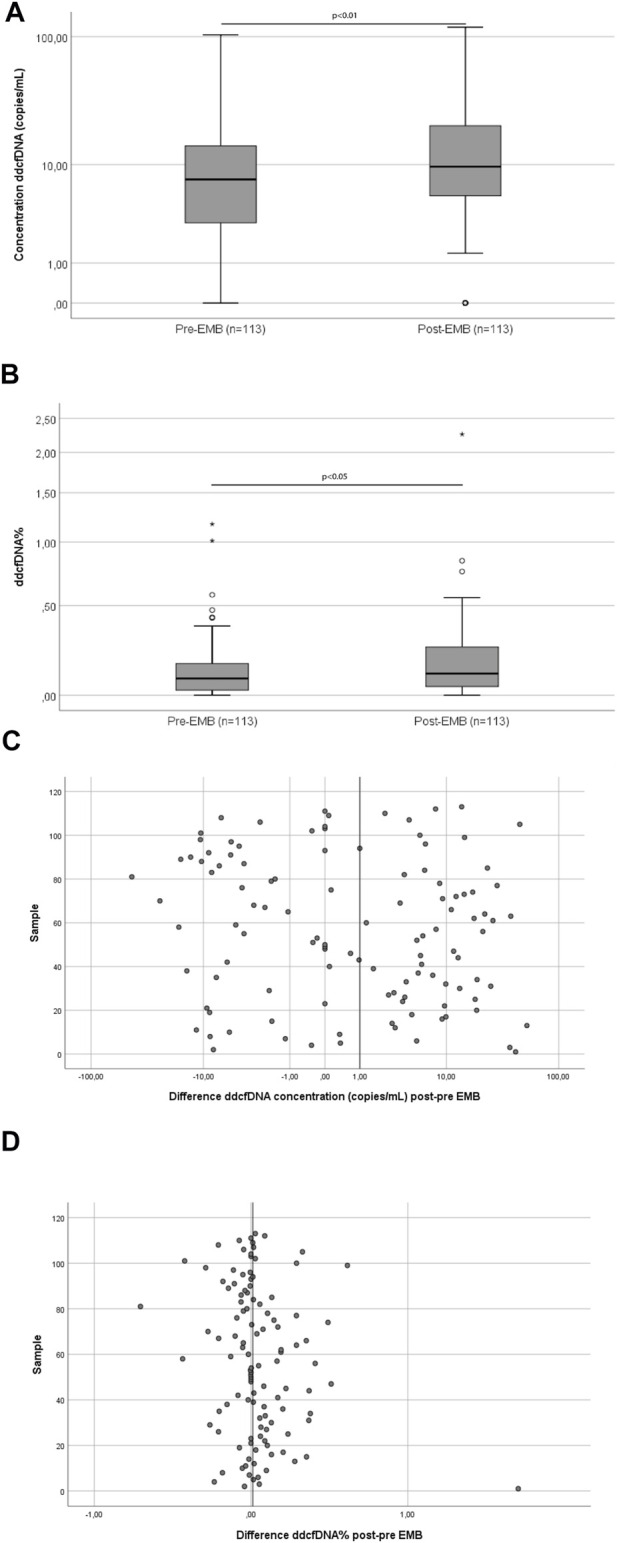
Pairwise comparison of ddcfDNA concentration **(A)** and ddcfDNA% **(B)** and absolute differences in ddcfDNA concentration **(C)** and ddcfDNA% **(D)** of samples taken before and after the EMB procedure. The middle line of the box represents the median and the upper and lower borders of the box represent the 25 and 75% percentile. Whiskers represent the 5th–95th percentile and the small circles represent outliers **(A,B)**. The vertical line on the x-axis represents the median differences **(C,D)**. Abbreviations: EMB, endomyocardial biopsy.

In order to assess the effect of the EMB procedure on ddcfDNA values, pre-EMB ddcfDNA values were compared with post-EMB values in the paired samples (*n* = 113). The median (IQR) pre-EMB ddcfDNA concentration was 7.5 (3.0–14.5) copies/ml. This concentration increased to 9.6 (5.4–20.8) copies/ml post-EMB, corresponding to a 1.28-fold increase (Figure 1A; *p* = 0.007). ddcfDNA% increased significantly from 0.08% (0.00–0.14) pre-EMB to 0.10% (0.02–0.20) post-EMB, corresponding to a 1.31-fold increase in ddcfDNA% (Figure 1B; *p* = 0.03). The absolute differences in ddcfDNA concentration and ddcfDNA% between pre- and post-EMB samples are represented in Figures 1C,D. There was no correlation between age (18–63 years) and fold change in both ddcfDNA% (*n* = 113; Spearman’s correlation coefficient *r* = −0.02, *p* = 0.74) and ddcfDNA concentration (*r* = −0.04, *p* = 0.64).

## Discussion

The present study was performed to assess the effect of the EMB procedure on plasma ddcfDNA values. We observed an increase in ddcfDNA concentration (1.28-fold) and ddcfDNA% (1.31-fold) in post-EMB samples, compared to pre-EMB samples. This illustrates that the EMB procedure causes iatrogenic injury to the allograft.

The EMB-related effect is mild in comparison with the effect of allograft rejection on ddcfDNA values as the reported differences in ddcfDNA values between acute rejection and non-rejection seem to be more pronounced; 0.17% during acute rejection and 0.07% during non-rejection, indicating a more than 2-fold increase in ddcfDNA% which is more than the 1.31-fold increase in ddcfDNA% in post-EMB samples (4).

The use of ddcfDNA as minimally invasive biomarker for acute rejection is meant to help clinicians determine whether it is necessary to perform an invasive EMB or not. This should reduce the amount of unnecessary EMBs in heart transplant recipients. However, despite the fact that the EMB procedure slightly increases ddcfDNA values in post-EMB samples, this effect could potentially still affect the evaluation of ddcfDNA as biomarker for allograft rejection in studies.

The currently published studies for acute rejection monitoring suggest threshold values for ddcfDNA% ranging from 0.15% to 2.0% (5). For example, a previous study suggested a threshold of 0.2%, with a corresponding sensitivity of 44% and a negative predictive value (NPV) of 97% for the detection of heart allograft rejection (4).

For the determination of a certain threshold value, the use of post-EMB samples could lead to inappropriately high suggested thresholds. An inappropriately high threshold means that the sensitivity of the assay decreases; more rejection episodes would be missed as the ddcfDNA values during these episodes are below the threshold that triggers for the performance of an EMB. In order to rule out such a potential effect of timing of sample collection on threshold values, samples thus need to be collected before an EMB procedure.

Another potential clinical application of ddcfDNA is to monitor the response of anti-rejection therapy within heart transplant recipients. A previous study showed that ddcfDNA% decreases after the start of anti-rejection therapy (17). To reliably examine a response of anti-rejection therapy, it is important that the ddcfDNA values are not affected by the EMB procedure. This is also a reason why samples need to be collected before an EMB procedure.

To the best of our knowledge, this is the first study that examined the effect of an EMB on ddcfDNA values in an adult heart transplant population. A previous publication of the effect of the EMB on ddcfDNA values in young heart transplant recipients observed a stronger EMB related increase in ddcfDNA which seemed to be age-dependent (18); a 35.1-fold increase in ddcfDNA concentration in pediatric patients and a 4.4 fold increase in young adults (aged 18–22 years) was observed (18). With respect to this age-dependent effect, the lower increase in the present study might be explained by a higher average age of the study population. Another explanation for the discrepancy between the results of these studies might be that both studies used different ddcfDNA quantification methods; the present used ddPCR, whereas ddcfDNA quantification in the previous study was performed by using quantitative real-time PCR. The time between the EMB and sample collection in both studies was similar and could therefore not be a reason for the observed discrepancy. This study had a limited amount of rejection episodes. Therefore, it was not possible to analyze ddcfDNA during rejection and non-rejection in these samples. In addition, there is no evidence that confounders such as rejection, infection, immunosuppressive therapy and time after transplantation influence the fold change induced by the EMB procedure. For a more robust analysis of these confounders, a larger cohort than that presented here, needs to be investigated.

The present study found that the EMB procedure affects both ddcfDNA% and ddcfDNA concentration alike as the fold increases in both were comparable (1.28-fold vs 1.31-fold). This illustrates that the EMB procedure itself does not cause fluctuations in recipient cfDNA.

To conclude, we observed an increase in ddcfDNA concentration and ddcfDNA% caused by iatrogenic injury occurring as a result of the EMB procedure. If ddcfDNA is to be a promising biomarker to detect allograft rejection in transplantation patients, it is important that this biopsy-related effect is taken into account. Collection of blood sampling before the EMB procedure is essential to prevent ddcfDNA values being affected by this procedure. The value of ddcfDNA concentration for rejection monitoring should be addressed in a future cohort with more rejection episodes.

## Data Availability

The raw data supporting the conclusion of this article will be made available by the authors, upon request.

## Supplementary Material

Supplementary Figure 1 Timing of the biopsies post-transplant with the corresponding pre-EMB ddcfDNA values and biopsy result. Figure 1A shows ddcfDNA concentration and Figure 1B shows ddcfDNA%. Abbreviations: ACR, acute cellular rejection.
