# Supplementary material for: Donor-Derived Cell-Free DNA for the Detection of Heart Allograft Injury: The Impact of the Timing of the Liquid Biopsy
Source: Transpl Int. 2022 Mar 21;35:10122. doi: 10.3389/ti.2022.10122 (PMC8977404; doi:10.3389/ti.2022.10122)
Supplement: Supplementary file 1 [file DataSheet1.docx]

# SUPPLEMENTARY FIGURE LEGENDS

**Supplementary Figure 1** Timing of the biopsies post-transplant with the corresponding pre-EMB ddcfDNA values and biopsy result. Figure 1A shows ddcfDNA concentration and Figure 1B shows ddcfDNA%. Abbreviations: ACR, acute cellular rejection.

**Figure S1A**

**
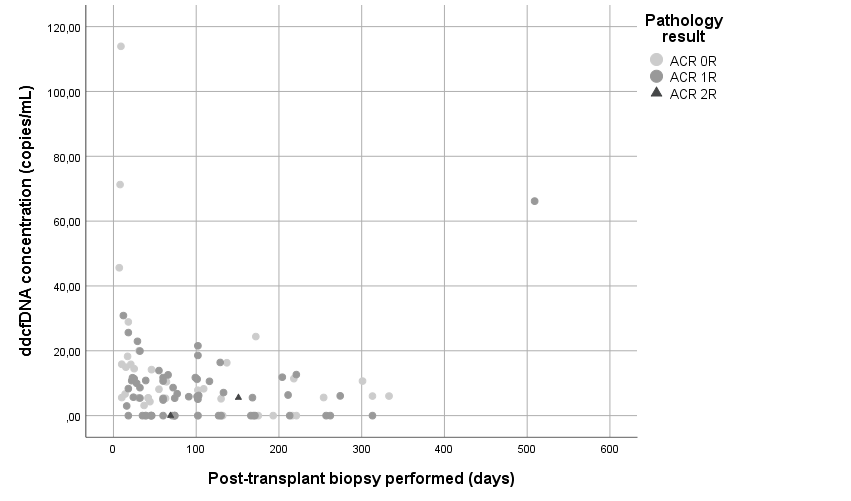
**

**Figure S1B**

**
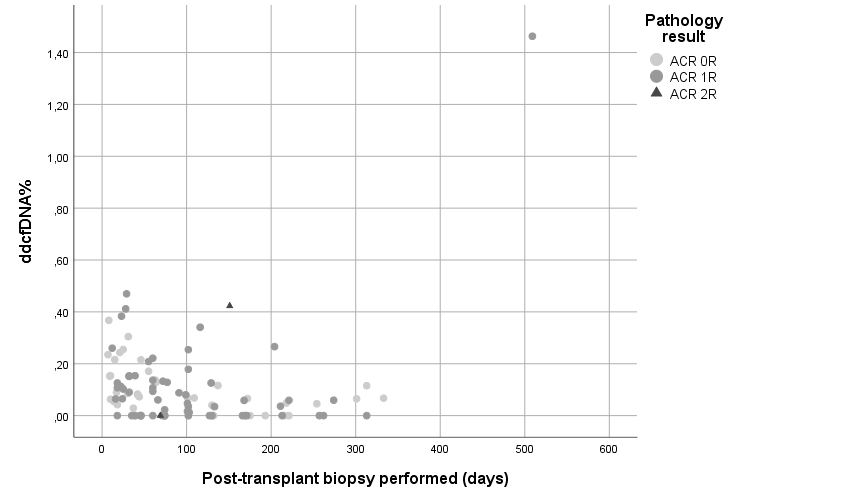
**
